# *Emm* type distribution of group A *Streptococcus* in China during 1990 and 2020: a systematic review and implications for vaccine coverage

**DOI:** 10.3389/fpubh.2023.1157289

**Published:** 2023-05-30

**Authors:** Caixin Xiang, Jianzhong Zhang, Fei Zhao, Yuanhai You

**Affiliations:** ^1^School of Public Health, China Medical University, Shenyang, Liaoning, China; ^2^State Key Laboratory for Infectious Disease Prevention and Control, National Institute for Communicable Disease Control and Prevention, Chinese Center for Disease Control and Prevention, Beijing, China

**Keywords:** group A *Streptococcus*, *emm* type, prevalent type, China, vaccine coverage

## Abstract

**Background:**

The recent increase of group A *Streptococcus* (GAS) infections in Europe has aroused global concern. We aim to provide molecular biological data for GAS prevention and control in China by analyzing the temporal shift of *emm* type.

**Methods:**

We collected studies reporting GAS *emm* types in China from 1990 to 2020 by PRISMA statement and established a summary database including *emm* types and literature quality assessment. Based on the database we analyzed the geographic distribution of *emm* types from 1990 to 2020 and assessed the coverage of the known GAS 30-valent vaccine. Outbreak-associated *emm* types that had been reported over the past 30 years were also included.

**Results:**

47 high quality studies were included for a systematic analysis of *emm* type distribution. This generated a database including totally 12,347 GAS isolates and 85 *emm* types. Shift of dominant *emm* type was witnessed during the past 30 years in China. In mainland China, dominant types changed from *emm*3, *emm*1, *emm*4, *emm*12 in 1990s to *emm*12 and *emm*1 in 2000s and 2010s. Hong Kong and Taiwan were dominated by *emm*12, *emm*4 and *emm*1, of which *emm*4 reduced but *emm*12 increased in 2010s significantly. From 1990 to 2020, newly found *emm* types were increasingly reported in various regions of China. The reported 30-valent M protein vaccine covered 26 M types prevalent in China, including all dominant types.

## Conclusion

*Emm*12 increased significantly in the past 30 years in China. The prevalent type of Hong Kong and Taiwan is slightly different from that of mainland China by *emm*4. Our long-term retrospective analysis implicates that the 30-valent M protein vaccine can cover all dominant *emm* types prevalent in the past 30 years in China, which provide important basic data for future vaccine evaluation.

## Introduction

Group A *Streptococcus* (GAS), also known as *Streptococcus pyogenes*, is an important gram-positive bacteria and one of the ten leading causes of infectious diseases in the world (1). GAS can cause wide spectrum of infections ranging from mild infections like pharyngitis and superficial skin infections, to serious and more deadly infections like invasive infections and streptococcal toxic shock syndrome (2). The larynx and epithelium of the skin are the primary sites for GAS infection and are also the common sites where GAS is acquired and transported (3). More than 18 million people worldwide are estimated to suffer from severe GAS disease (4). The estimated annual incidence of GAS caused pharyngitis is 0.4 cases per person-year, with more than 423 million children infected with GAS in developing countries (1). Although the most common infection is pharyngitis, invasive disease has increased dramatically in many areas of the world over the past 25 years (5). With the situations that many countries have co-existed with COVID-19, resurgence of group A *Streptococcus* infections has been noted in the last months in European countries.

M protein is a major virulence determinant and protective antigen of GAS. *Emm*, the gene encoding M protein, is the basis for sequence typing that used to differentiate strain serotype. *Emm* typing is based on sequence diversity of the 5′ region of *emm* (6–9). Sequence diversity of M protein has generated over 260 different *emm* types currently reported in the database from US CDC. *Emm* genotyping can provide basic information for possible vaccine implementation (10) and long-term surveillance of *emm* types is very important for detection of emerging types. It is always a challenge to develop a safe vaccine against group A *Streptococcus* infection (11). Recently a recombinant 30-valent M protein vaccine has passed Phase I clinical trial and could be potentially safe and effective for prevention of GAS infection (12). However, the molecular epidemiological data for GAS circulating in various geographies of the world is insufficient, which could limit further evaluation and utilization of a vaccine. In a study reported on global *emm* type distribution, little data was available from China and it is also in need to update the molecular data (13).

China has always attached great importance on the management and prevention of group A *Streptococcus* diseases. Research on GAS molecular epidemiology began in the early 1990s (14). Many studies from different geographies of China have reported collections of large number of GAS isolates and *emm* genotypes (15–19), there is a tremendous need to make a systematic review and summarization on these studies. In this study we collected and reviewed all reports on *emm* genotyping of China GAS isolates during the past 30 years. We aim to provide comprehensive *emm* data for GAS prevention and control, as well as to assess the coverage of 30-valent M protein vaccine for China.

## Materials and Methods

### Data sources

**Figure 1:**
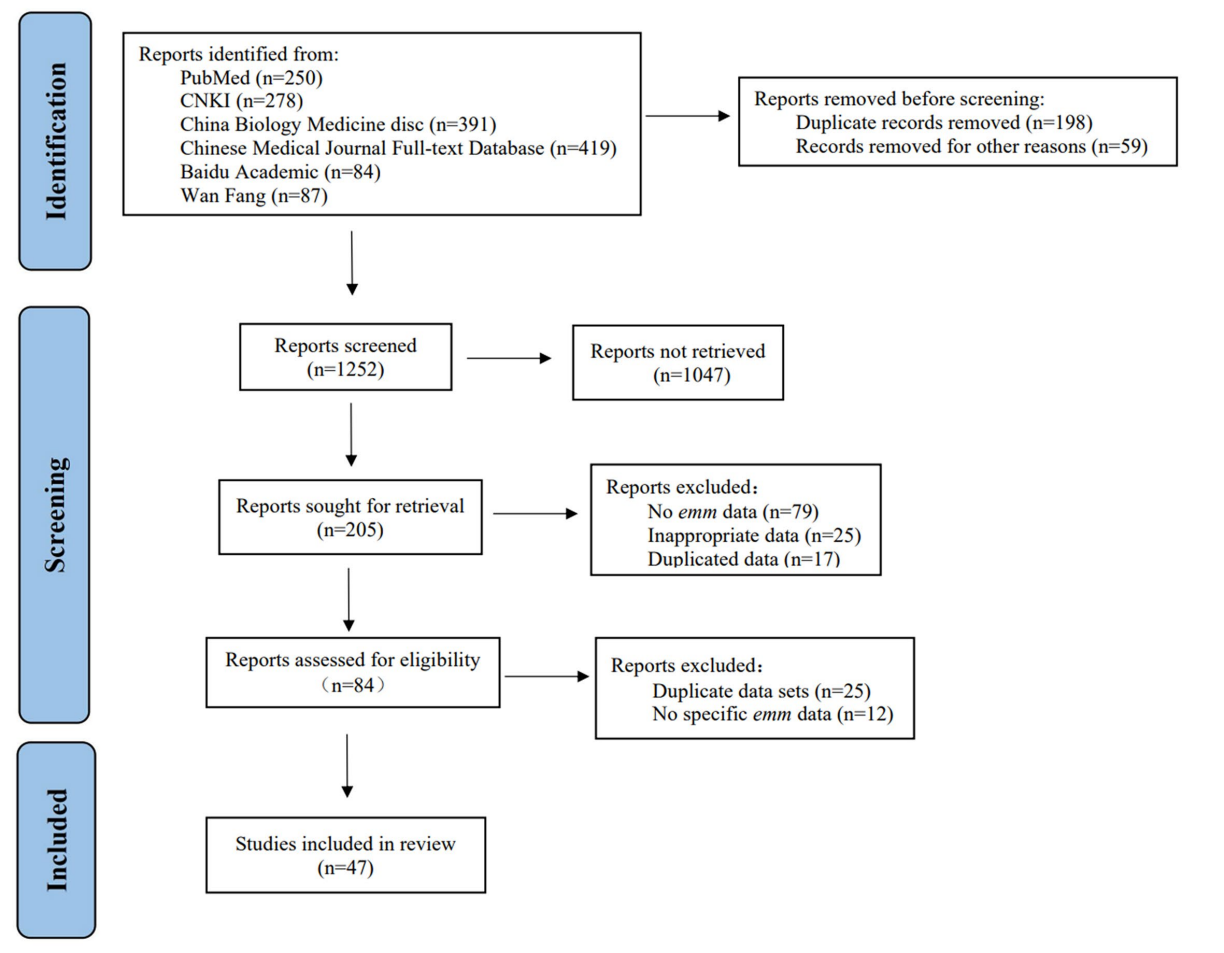
Flow chart of literature collection and screening.

Studies describing the molecular epidemiology of GAS based on *emm* or M typing were searched by using a systematic approach consistent with the PRISMA statement (20). The search process was shown in Figure 1. We searched for publications involved in GAS genotyping and molecular epidemiology published from January 1, 1990 to June 30, 2021, with the search updated on May 26, 2022. The language was limited to Chinese and English.

Chinese databases (CNKI, Wan Fang, China Biology Medicine disc, Chinese Medical Journal Full-text Database, Baidu Academic) and English database (PubMed) were searched. Search terms included “genotype,” “genotyping,” “group A streptococci,” “group A *Streptococcus*,” “*Streptococcus pyogenes*,” “GAS,” “*emm*,” “molecular typing,” “molecular epidemiology,” etc. (Supplementary Appendix S1).

### Study quality assessment

We established a database that dedicated to aggregated data on results about *emm* genotype of GAS. The contents of the database include study time, region, population, *emm* typing method, etc., in order to evaluate the data and its eligibility for inclusion. Regarding data extraction and citation, two researchers screened the literature (Supplementary Appendix S1, S2).

In order to make an overall estimation of all GAS genotypes in China, we included studies of mainland, Taiwan, and Hong Kong. Macau was excluded since there were few reported cases of GAS. When there was duplicate data (for example, multiple studies reporting data from the same region or at the same time, or using the same strains), we included only the most recent study and remove repeated reports.

### Definition

To evaluate the time and regional distribution characteristics of China GAS *emm* types, the regions were divided into mainland, Hong Kong and Taiwan, and the time was divided into three periods: the 1990s, 2000s and 2010s.

A 30-valent GAS vaccine based on M protein had been reported, including *emm*1, 2, 3, 4, 5, 6, 11, 12, 14, 18, 19, 22, 24, 28, 29, 44, 49, 58, 73, 75, 77, 78, 81, 82, 83, 87, 89, 92, 114, 118 (12). Vaccine coverage was defined as the percentage of the number of *emm* genotypes covered by the reported 30-valent GAS vaccine in a given region to the total number of *emm* genotypes in the region. Vaccine coverage for 10 most common types was the extent to which the 30-valent vaccine can cover the 10 most common GAS *emm* genotypes in a region. It was calculated as the number of *emm* genotypes included in the 30-valent vaccine among the 10 most common *emm* genotypes in each region divided by 10 and then multiplied by 100%. Vaccine coverage of the dominant genotype referred to the percentage of the ratio of the number of dominant genotypes to the number of dominant genotypes covered by the 30-valent vaccine. If the number of strains of a certain *emm* type accounted for more than 10% of all strains, it was regarded as a dominant genotype.

GAS outbreak study was defined as the first reported outbreak study on the *emm* genotype of GAS in a certain region. GAS case study was defined as the newly discovered or first reported case study on the *emm* genotype of GAS in a certain region.

### Statistical analysis

By sorting the percentages of different *emm* genotypes of GAS strains in the total number of GAS strains in the designated area, we compared the distribution of *emm* types in different regions of China. We also compared the distribution of *emm* types in different time periods by the same method. Excel 2019 was used to establish the database and conduct data analysis.

## Results

### Diversity of GAS *emm* types in China

We retrieved 1,509 documents in specific databases according to the search method and search terms. We removed duplicate literatures. After preliminary screening of titles and abstracts, 1,304 literatures did not meet the research content were removed and the remaining 205 full-text literatures were kept for further analysis. Furthermore, we excluded 158 articles by quality assessment on the 205 published datasets (Figure 1). Finally, 47 published high-quality articles were included for qualitative analysis (Supplementary Appendix S1), including 9 articles on GAS outbreaks and case studies about *emm* genotypes in China. Totally 54 datasets were included in our database, including 36 datasets for mainland, 14 datasets for Taiwan, and 4 datasets for Hong Kong (Table 1).

**Table 1:** Summary of *emm* types of GAS strains in China.

Area	The data sets	GAS strain (number)	*Emm* types
Mainland	36	8,820	60
Hong Kong	4	483	51
Taiwan	14	3,044	47
Total	54	12,347	85

We pooled 12,347 GAS strains related to 85 *emm* genotypes (Table 1). The most popular *emm* genotypes are *emm*12 (50.08%), *emm*1 (30.47%), *emm*4 (6.31%), *emm*22 (1.98%), *emm*6 (1.91%), accounting for 90.75% of all strains (Supplementary Appendix S2).

### Temporal distribution of *emm* types

**Figure 2:**
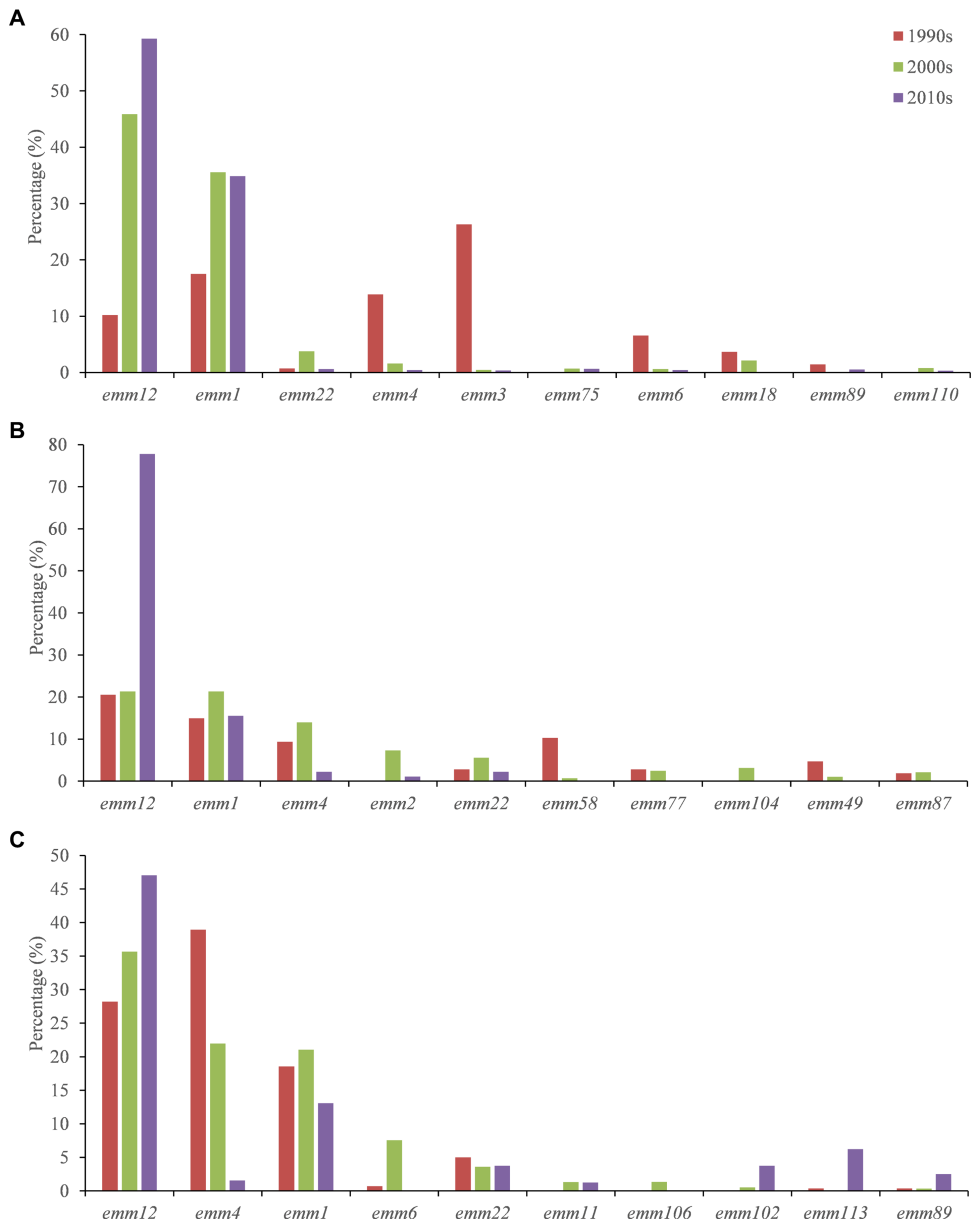
Temporal distribution of *emm* types of GAS strains in mainland **(A)**, Hong Kong **(B)** and Taiwan **(C)**.

In the mainland, compared with the period of 1990s, strains of *emm*1, *emm*12 and *emm*22 increased significantly in the 2000s, whereas *emm*3, *emm*4 and *emm*6 decreased significantly. Compared with the 2000s, *emm*1 showed little change in the 2010s, whereas *emm*12 significantly increased. In Hong Kong, *emm*1, *emm*2, *emm*4 and *emm*22 increased in the 2000s compared with the period of 1990s, but *emm*58 decreased significantly. *Emm*12 did not change much. Compared with the 2000s, *emm*2 and *emm*4 GAS strains decreased significantly in the 2010s, but *emm*12 increased significantly. In Taiwan, compared with the period of 1990s, *emm*1, emm6 and *emm*12 increased significantly in the 2000s, whereas *emm*4 decreased significantly. Moreover, *emm*12 had been increasing over the past 30 years, while *emm*4 had been decreasing (Figure 2).

### Regional distribution of *emm* types

**Figure 3:**
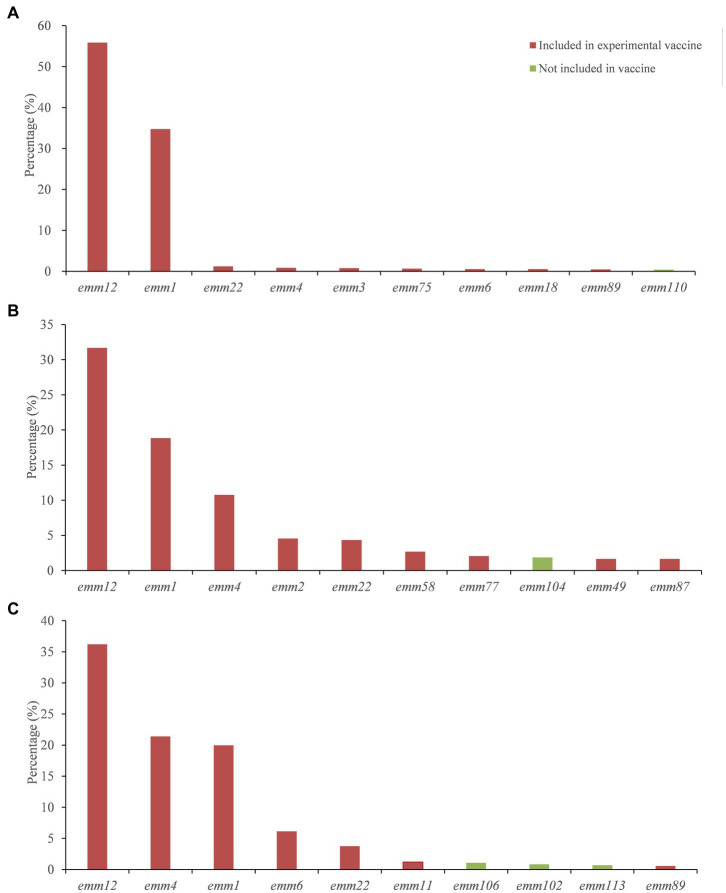
*Emm* types among the top 10 covered by the 30-valent M protein vaccine in mainland **(A)**, Hong Kong **(B)** and Taiwan **(C)**.

The distribution of GAS *emm* genotypes in different regions of China was different (Supplementary Appendix S2). We aggregated 8,820 strains and contained 60 *emm* genotypes in mainland region (Table 1). *Emm*12 (55.87%) and *emm*1 (34.73%) were the dominant genotypes, followed by *emm*22 (1.25%). Figure 3A listed 10 common *emm* genotypes of GAS strains in the mainland, which accounted for 96.11% of the GAS strains in the mainland. The remaining 50 *emm* genotypes accounted for 2.63% of the GAS strains in the mainland.

A total of 483 strains were collected in Hong Kong, including 51 *emm* genotypes (Table 1). *Emm*12 (31.68%), *emm*1 (18.84%) and *emm*4 (10.77%) were the dominant genotypes, accounting for 61.29% of the *emm* genotypes of the GAS strains included in Hong Kong. Figure 3B listed 10 common *emm* genotypes of GAS strains in Hong Kong, which accounted for 80.13% of all GAS strains. The remaining 41 *emm* genotypes accounted for 18.01% of all GAS strains in Hong Kong.

In Taiwan, 3,044 strains were collected, including 47 *emm* genotypes (Table 1). *Emm*12 (36.2%), *emm*4 (21.39%) and *emm*1 (19.97%) were the dominant genotypes, accounting for 77.56% of the *emm* genotypes of the GAS strains included in Taiwan. Figure 3C listed 10 common *emm* genotypes of GAS strains in Taiwan, which accounted for 91.78% of GAS strains in Taiwan. The remaining 37 *emm* genotypes accounted for 4.7% of all GAS strains in Taiwan.

### Assessment of 30-valent experimental vaccine coverage

The 30-valent M protein vaccine covered 26 M types prevalent in China (21, 20, and 21 *emm* genotypes in mainland, Hong Kong and Taiwan, respectively). Table 2 summarized the coverage of international 30-valent GAS M protein vaccine of the Chinese GAS strain *emm* genotypes by region. Overall, the vaccine coverage in mainland, Taiwan and Hong Kong was low, however vaccine coverage for the 10 most common types and the dominant genotypes were very high (Figure 3).

**Table 2:** Vaccine coverage by region in China.

Area	*Emm* type(s) included in 30-valent M protein vaccine	Overall vaccine coverage (%)	Vaccine coverage for 10 most common types (%)	Vaccine coverage of the dominant genotype (%)
Mainland	1, 2, 3, 4, 5, 6, 11, 12, 18, 22, 28, 44, 49, 58, 75, 77, 81, 82, 87, 89, 114	35.0	90	100
Hong Kong	1, 2, 3, 4, 11, 12, 14, 18, 22, 28, 49, 58, 73, 75, 77, 81, 82, 87, 89, 118	39.22	90	100
Taiwan	1, 2, 3, 4, 6, 11, 12, 22, 28, 44, 49, 58, 73, 75, 77, 78, 81, 82, 87, 89, 92	44.68	70	100
Total	1, 2, 3, 4, 5, 6, 11, 12, 14, 18, 22, 28, 44, 49, 58, 73, 75, 77, 78, 81, 82, 87, 89, 92, 114, 118	30.59	–	–

### *Emm* types involved in outbreaks and case studies

We also summarized GAS *emm* genotypes from outbreaks and case studies in China from 1990 to 2020. The results were shown in Table 3. In the past 30 years, GAS outbreaks caused by different *emm* types had occurred in various regions of China. In 2005, acute glomerulonephritis (AGN) outbreaks occurred in two counties in Guizhou Province, which were caused by GAS *emm*60.1 and *emm*63.0 (21). In 2006, an outbreak of adult scarlet fever caused by the GAS *emm*75 genotype occurred in Zhejiang Province (22). In 2012, the first outbreak of tonsillopharyngitis caused by GAS *emm*89 genotype occurred in Beijing (23), in the same year an outbreak of scarlet fever caused by the *emm*1 and *emm*12 genotypes occurred in Zhejiang Province (24). In 2013, an outbreak of GAS caused by the *emm*5 genotype occurred in Shanghai Province, which was the first GAS outbreak in the workplace (25).

**Table 3:** Summary of *emm* types in GAS outbreaks and case studies in China from 1990 to 2020.

Year	Province/City	*Emm* type	Definition	Note
2003	Hong Kong	*emm*1	First case of primary GAS psoas abscess	Case
2005	Guizhou	*emm*60.1, *emm*63.0	Outbreaks of AGN	Outbreak
2006	Guangdong	stg485	Case of rheumatic fever	Case
2006	Zhejiang	*emm*75	An outbreak of scarlet fever in adults	Outbreak
2012	Beijing	*emm*89	First outbreak of GAS *emm* 89 tonsil pharyngitis	Outbreak
2012	Zhejiang	*emm*1, *emm*12	An outbreak of scarlet fever	Outbreak
2013	Shanghai	*emm*5	First workplace GAS outbreak	Outbreak
2015	Beijing	*emm*89.0	First case of adult necrotizing fasciitis with toxic shock syndrome	Case
2017	Hunan	*emm*1.1	A case of toxic shock syndrome caused by GAS	Case

In the past 30 years in China, there were not only several new GAS genotypes that were unreported previously, but also more and more unusual GAS infections were reported. In 2003, the first case of primary GAS psoas abscess caused by *emm*1 was found in Hong Kong (26). In 2006, a patient with rheumatic fever caused by GAS *emm* stg485 was found in Guangdong Province (27). In 2015, Chinese first case of adult necrotizing fasciitis with toxic shock syndrome caused by *emm*89.0 was reported in Beijing (28). A case of toxic shock syndrome caused by GAS *emm*1.1 occurred in Hunan Province in 2017 (29). The findings of these reports emphasize the importance of surveillance of invasive GAS infection in China.

## Discussion

By using qualitative analysis on all included literatures and excluding strains that could not be typed, totally 85 *emm* types were found for China GAS strains in the past 30 years, which indicated that *emm* genotypes of China GAS were diverse. The most prevalent genotype was *emm*12, accounting for 50.08% of those included in this study, followed by *emm*1 (30.47%) and *emm*4 (6.31%). This is different from the situation in Europe and North America, where *emm*1 is the dominant type, occasionally replaced by other *emm* types (30). More importantly, the emergent M1_UK_ clone in Europe accounted for the majority of increasing scarlet fever and invasive infection cases in the past years (31), which suggests that genomic epidemiologic surveillance is in urgent need in China to screen the transmission of such virulent clones on time.

In the past 30 years, not only some new GAS genotypes and new types of infections that were never reported previously appeared in various regions of China, but also many GAS outbreaks caused by different *emm* genotypes had occurred. These results suggest that there are potentially other unknown *emm* genotypes that were not identified in China. Therefore, it is necessary to perform long-term molecular epidemiological monitoring of GAS genotypes. Especially under the context of COVID-19 epidemic, introduction of novel *emm* types could probably trigger more GAS outbreaks hence increase medical burden.

There were different *emm* genotypes of GAS prevalent in different times and regions in China during the past 30 years. In particular, the molecular epidemiology and shift of GAS *emm* pattern in mainland China, Taiwan and Hong Kong were not exactly consistent. In mainland China during the past 30 years, *emm*1 and *emm*12 had been the dominant types since 2000s, with the percentage of *emm*1 increasing from 17.52% in the 1990s to 35.55% in the 2000s, and the percentage of *emm*12 increasing from 10.22% in the 1990s to 45.89% in the 2000s (Supplementary Appendix S1). Lu et al. collected 140 *S. pyogenes* from infected patients in 10 tertiary general hospitals from 7 cities/provinces in China during 2009 and 2016, and found that *emm* 12 and *emm*1 had a relatively high proportion among all isolates (32). Compared with the period of 2000s, the percentage of *emm*1 GAS strains in the 2010s did not change too much from 35.55% to 34.86%, but *emm*12 increased significantly from 45.89% to 59.28% (Supplementary Appendix S1). There are regional differences between various cities or provinces of mainland. Although *emm*12 and *emm*1 were the dominant GAS types in Beijing, *emm*12 ratio decreased from 76.4% in 2011 to 46.49% in 2020–2021 and *emm*1 ratio increased from 17.1% (2011) to 25.44% (2020–2021), respectively (16, 18). *Emm*12 was also the predominant circulating type (91.4%) in Shanghai in 2011 (15). Meanwhile, *emm*12 took up to 60.3% in fluoroquinolone non-susceptible GAS during 2011 to 2016 in Shanghai (17). The outbreak and resurgence of group A streptococcal disease such as scarlet fever is probably due to the emergence of a newly found macrolide resistant *emm*12 clone in 2011 (15, 17–19, 33). You et al. conducted genomic studies on *emm*12 strains and found several mobile genetic elements that are associated with the scarlet fever outbreak in recent years. Such as ICE-*emm*12 which encoding macrolide and tetracycline resistance genes, and the prophage φHKU.vir encoding virulence factors *SSA*, *SpeC* and *Spd1* (34). Chen et al. found that *emm*1 GAS strains in Shanghai during 2011–2015 harbored a superantigen profile similar to *emm*12 isolates, except for the *SpeA* gene (35). These studies could partially explain the increase of *emm*1 and *emm*12 GAS strains in the mainland over the last 30 years though more detail studies are needed.

In Taiwan and Hong Kong, the constitution of prevalent *emm* types in the past thirty years is different. Ten most common *emm* genotypes in Taiwan are *emm*12, *emm*4, *emm*1, *emm*6, *emm*22, *emm*11, *emm*106, *emm*102, *emm*113, *emm*89. Chiang-Ni et al. collected 677 isolates from 1994 to 2008 in a hospital in southern Taiwan. They found that forty-four different *emm* types, the five most frequent types were *emm*12*, emm*4*, emm*1*, emm*11 *and emm*81, which were responsible for 61.4% of all isolates (36). In Hong Kong, the 10 most common types were *emm*12, 1, 4, 2, 22, 58, 77, 104, 49, 87, which were different from the mainland and Taiwan except for the most dominant types *emm*12, *emm*1, and *emm*4. The reasons for *emm* pattern difference and diversity between mainland, Taiwan and Hong Kong remain unclear. More comprehensive surveillance data is needed for further clarification. Our findings also have implications for vaccine development and evaluation. M protein can elicit bactericidal antibodies following GAS infection that persist in human serum for extended periods of time. There is a 30-valent M protein vaccine reported safe and immunogenic for humans, and has passed Phase I clinical trials (12). Our results showed that the 30-valent vaccine covered 26 GAS *emm* genotypes in China. Although the overall genotype coverage in China was low, the coverage of 10 most common types and the dominant genotypes were very high.

There are some limitations for our study. Firstly, our data did not cover all provinces of China, especially those western provinces where little data is available, this could potentially affect the diversity of *emm* types found in this study, which emphasize the importance of sampling and GAS genotyping in these areas. Secondly, most of the included studies were conducted during 2000s and 2010s. Few data are available in 1990s. Therefore, the qualitative comparison results obtained in this study need to be interpreted with caution.

In conclusion, according to the available data from various regions in China, the dominant *emm* types between mainland China, Hong Kong and Taiwan are similar, but shifted in different time period. *Emm*12 and *emm*1 are the two most important types that need to be highly concerned in future surveillance. The experimental 30-valent M protein vaccine provides good coverage on dominant *emm* genotypes in China, which could be useful for the prevention and control of GAS infection in China.

## Author contributions

CX collected data and made systematic review. CX and YY write the manuscript. JZ and FZ help review data and revise the manuscript. All authors contributed to the article and approved the submitted version.

## Funding

This study was supported by Surveillance and epidemic response of bacterial and fungal infectious diseases (08025) and Project for Novel Detection Techniques of Bacterial Pathogens (32073).

## Conflict of interest

The authors declare that the research was conducted in the absence of any commercial or financial relationships that could be construed as a potential conflict of interest.

## Publisher’s note

All claims expressed in this article are solely those of the authors and do not necessarily represent those of their affiliated organizations, or those of the publisher, the editors and the reviewers. Any product that may be evaluated in this article, or claim that may be made by its manufacturer, is not guaranteed or endorsed by the publisher.
