# Supplementary material for: Emm type distribution of group A Streptococcus in China during 1990 and 2020: a systematic review and implications for vaccine coverage
Source: Front Public Health. 2023 May 30;11:1157289. doi: 10.3389/fpubh.2023.1157289 (PMC10262998; doi:10.3389/fpubh.2023.1157289)
Supplement: Supplementary file 1 [file Data_Sheet_1.docx]

Appendix 1

1. Combined search strategy for *emm* types in China (1990-2020): PubMed.

Date of last search: 2022.5.20

Database: PubMed

Filters: 1990-2021

|  | PubMed |
| --- | --- |
| 1(MESH) | *Streptococcus* *pyogenes* OR group A *Streptococcus* |
| 2(MESH) | Genotype OR *emm* type OR typing OR *emm* OR M protein |
| 3(MESH) | China OR Hong Kong OR Taiwan |
| 4(MESH) | Molecular epidemiology OR Outbreak |
| 5(MESH) | Scarlet fever |
| 6(MESH) | (Group A strep* OR strep* group A* OR step* pyogenes) |
| 7 | 1 OR 6 |
| 8 | 4 OR 5 |
| 9 | 7 AND 2 AND 3 (109 articles) |
| 10 | 7 AND 8 AND 3 (141 articles) |

2. Search strategy for *emm* types in China (1990-2020): CNKI and Chinese Medical Journal Full-text Database.

Date of last search:2022.5.26

Databases: CNKI, Chinese Medical Journal Full Text Database

Filters: 1990.01.01-2021.06.30

|  | CNKI | Chinese Medical Journal Full-text Database |
| --- | --- | --- |
| 1 | Different terms of group A *Streptococcus* in Chinese | Different terms of group A *Streptococcus* in Chinese |
| 2 | Genotype OR gene typing | (419 articles) |
| 3 | Pathogen |  |
| 4 | 1 AND (2 OR 3) （278 articles） |  |

3. Search strategy for *emm* types in China (1990-2020): China Biology Medicine disc, Baidu Academic, Wan Fang.

Date of last search: 2022.5.26

Databases: China Biology Medicine disc, Baidu Academic, Wan Fang

Filters: 1990-2021

|  | China Biology Medicine disc | Baidu Academic | Wan Fang |
| --- | --- | --- | --- |
| 1 | Different terms of group A *Streptococcus* in Chinese | Different terms of group A *Streptococcus* in Chinese | Different terms of group A *Streptococcus* in Chinese |
| 2 | Molecular epidemiology OR genotype OR gene typing OR *emm* OR pathogen | Molecular epidemiology OR genotype OR gene typing OR *emm* OR pathogen | Molecular epidemiology OR genotype OR gene typing OR *emm* OR pathogen |
| 3 | 1 AND 2(391 Chinese articles） | 1 AND 2（84 articles） | Scarlet fever |
| 4 |  |  | 1 AND 2 AND 3(87 articles) |

4. Study selection criteria.

| Criteria |
| --- |
| Eligibility criteria  All GAS strains should be collected from China between 1990 and 2020, including Hong Kong and Taiwan. The study should include strains that newly discovered and the first reported genotypes. |
| Exclusion criteria  Publication type  Articles without full text  Duplicate publication  Guidelines  Study type  Not China studies  No *emm* data or specific *emm* data studies  No detailed strain background  Duplicate data studies  Typing  All GAS isolates are characterized by *emm* type according to the standard protocol of Centers for Disease Control and Prevention (CDC; <http://www.cdc.gov/streplab/protocol-emmtype.html>). |

5. Quality assessment of included studies

| Author  (Publication) | Study time | Sample size | Typing | Data  Integrity | Data  diversity | Total |
| --- | --- | --- | --- | --- | --- | --- |
| (Li et al., 2020b) | 1 | 1 | 1 | 1 | 1 | 5 |
| (Li et al., 2020a) | 1 | 1 | 1 | 1 | 0 | 4 |
| (You et al., 2020) | 1 | 1 | 1 | 1 | 1 | 5 |
| (Ji et al., 2012) | 1 | 0 | 1 | 1 | 0 | 3 |
| (Liang et al., 2010) | 1 | 1 | 1 | 1 | 0 | 4 |
| (Yin et al., 2019) | 1 | 1 | 1 | 1 | 0 | 4 |
| (Liu, 2015) | 1 | 1 | 1 | 1 | 0 | 4 |
| (Bai et al., 2019) | 1 | 1 | 1 | 1 | 0 | 4 |
| (Wang et al., 2020) | 1 | 0 | 1 | 1 | 0 | 3 |
| (Hong et al., 2021) | 1 | 0 | 1 | 1 | 0 | 3 |
| (Shen et al., 2018) | 1 | 1 | 1 | 1 | 1 | 5 |
| (Zhang et al., 2019) | 1 | 0 | 1 | 1 | 0 | 3 |
| (Ouyang et al., 2021) | 1 | 1 | 1 | 1 | 0 | 4 |
| (Yang and Wang, 2020) | 1 | 1 | 1 | 1 | 0 | 4 |
| (Chen et al., 2008) | 1 | 0 | 1 | 1 | 0 | 3 |
| (Li et al., 2009) | 1 | 0 | 1 | 1 | 0 | 3 |
| (Zeng et al., 2016) | 1 | 0 | 1 | 1 | 1 | 4 |
| (Tan et al., 2019) | 1 | 0 | 1 | 1 | 0 | 3 |
| (Yu et al., 2021) | 1 | 1 | 1 | 1 | 1 | 5 |
| (Li et al., 2018) | 1 | 1 | 1 | 1 | 0 | 4 |
| (Li et al., 2010) | 1 | 0 | 1 | 1 | 1 | 4 |
| (Jing et al., 2006) | 1 | 0 | 1 | 1 | 1 | 4 |
| (Ma et al., 2009) | 1 | 1 | 1 | 1 | 1 | 5 |
| (Feng et al., 2010) | 1 | 1 | 1 | 1 | 1 | 5 |
| (Chang et al., 2011) | 1 | 1 | 1 | 1 | 1 | 5 |
| (Liang et al., 2012) | 1 | 1 | 1 | 1 | 1 | 5 |
| (You et al., 2013) | 1 | 0 | 1 | 1 | 0 | 3 |
| (Ho et al., 2003) | 1 | 1 | 1 | 1 | 1 | 5 |
| (Chan et al., 2009) | 1 | 1 | 1 | 1 | 1 | 5 |
| (Luk et al., 2012) | 1 | 0 | 1 | 1 | 0 | 3 |
| (Yan et al., 2003) | 1 | 0 | 1 | 1 | 0 | 3 |
| (Huang et al., 2014) | 1 | 1 | 1 | 1 | 1 | 5 |
| (Chen et al., 2007) | 1 | 1 | 1 | 1 | 1 | 5 |
| (Lin et al., 2011) | 1 | 0 | 1 | 1 | 1 | 4 |
| (Chiang-Ni et al., 2021) | 1 | 1 | 1 | 1 | 1 | 5 |
| (Chiou et al., 2004) | 1 | 1 | 1 | 1 | 0 | 4 |
| (Chiou et al., 2009) | 1 | 1 | 1 | 1 | 1 | 5 |
| (Tsai et al., 2021) | 1 | 1 | 1 | 1 | 1 | 5 |
| (Lau et al., 2003) | 1 | 0 | 1 | 1 | 0 | 3 |
| (Zheng et al., 2009) | 1 | 0 | 1 | 1 | 1 | 4 |
| (Huang et al., 2006) | 1 | 0 | 1 | 1 | 0 | 3 |
| (Dong et al., 2007) | 1 | 0 | 1 | 1 | 0 | 3 |
| (Liu et al., 2014) | 1 | 0 | 1 | 1 | 0 | 3 |
| (Xu et al., 2016) | 1 | 0 | 1 | 1 | 0 | 3 |
| (Chen et al., 2017) | 1 | 0 | 1 | 1 | 0 | 3 |
| (Wang et al., 2016) | 1 | 0 | 1 | 1 | 0 | 3 |
| (Ning et al., 2017) | 1 | 0 | 1 | 1 | 0 | 3 |

+Scoring criteria for included studies:

1. Study time is between 1990 and 2020. 1 point

2. Adequate sample strains (>100). 1 point

3. All isolates were characterized by *emm* type according to the standard protocol of Centers for Disease Control and Prevention (CDC; <http://www.cdc.gov/streplab/protocol-emmtype.html>). 1 point

4. Study data described in detail. 1 point

5. Diversity of data: the number of GAS *emm* genotypes (≥10). 1 point

5.Characteristics of the included studies.

| Author  (Publication) | Study period | Definition | Region | Sources | Isolated  strains | Types |
| --- | --- | --- | --- | --- | --- | --- |
| (Li et al., 2020b) | 2019.1-12 | Patients who were suspected to have GAS infection | Beijing | Throat swab, skin swab | 271 | 13 |
| (Li et al., 2020a) | 2016.01-2017.12 | Pediatric patients with scarlet fever | Beijing | Throat swab | 297 | 9 |
| (You et al., 2020) | 2011-2018 | Patients with scarlet fever or streptococcal infection | Beijing | Throat swab | 2484 | 21 |
| (Ji et al., 2012) | 2003-2008 | Pediatric patients with impetigo | Beijing | Skin swab | 52 | 7 |
| (Liang et al., 2010) | 2007 | Children with scarlet fever, pharyngotonsillitis or healthy carriers with GAS | Beijing | Throat swab | 155 | 8 |
| (Yin et al., 2019) | 2011-2016 | Children with scarlet fever | Tianjin | Throat swab | 189 | 5 |
| (Liu, 2015) | 2013-2014 | Children with scarlet fever, pharyngitis, or asymptomatic carriers with GAS | Shandong | Throat swab | 154 | 5 |
| (Bai et al., 2019) | 2012-2018 | Healthy people, scarlet fever patients and close contacts | Shandong | Throat swab | 143 | 5 |
| (Wang et al., 2020) | 2017 | Scarlet fever patients and close contacts | Shandong | Throat swab | 21 | 2 |
| (Hong et al., 2021) | 2016-2018 | Not stated | Jiangsu | Pharyngeal swab, nasal swab, pus and secretion, sputum | 50 | 5 |
| (Shen et al., 2018) | 2011.06-2016.06 | A S. *pyogenes* isolate database representative of the Shanghai population | Shanghai | NA | 2258 | 16 |
| (Zhang et al., 2019) | 2018.01-12 | Pediatric outpatients who were clinically diagnosed as scarlet fever | Shanghai | Throat swab | 86 | 7 |
| (Ouyang et al., 2021) | 2019.01-12 | Children from scarlet fever surveillance sites in Minhang District, Shanghai | Shanghai | Throat swab | 179 | 4 |
| (Yang and Wang, 2020) | 2017.01-2018.01 | Children with scarlet fever | Zhejiang | Throat swab | 147 | 5 |
| (Chen et al., 2008) | 2005.01-2006.12 | Children with respiratory infections, septic skin infections, or scarlet fever | Guangdong | Throat swab, skin swab | 87 | ＞7 |
| (Li et al., 2009) | 2007.01-2009.12 | Children with acute pharyngitis, acute tonsillitis, acute rheumatic fever, acute glomerulonephritis, or other GAS infections | Guangdong | Throat swab, skin swab | 98 | ＞8 |
| (Zeng et al., 2016) | 2013.02-2015.11 | Children with acute pharyngitis, acute tonsillitis, rheumatic fever, or scarlet fever | Guangdong | Throat swab, skin swab | 76 | ＞9 |
| (Tan et al., 2019) | 2017 | Children with scarlet fever | Guangdong | Throat swab | 43 | 3 |
| (Yu et al., 2021) | 2016-2018 | children aged < 18 years who were admitted to Shenzhen Children’s Hospital for treatment of one of 15 diseases | Guangdong | Throat swabs, sputum samples, pus samples, wound secretions, vulvar secretions, blood samples, urine sample | 342 | 10 |
| (Li et al., 2018) | 2014.01-2017.06 | Children with acute post-streptococcal glomerulonephritis | Henan | Throat swab | 113 | 7 |
| (Li et al., 2010) | 2007 | Children with pharyngeal diseases | Chongqing | Throat swab | 44 | 10 |
| (Jing et al., 2006) | 2000-2004 | Patients in different regions of mainland China | China | Blood, epipharynx, pus, wounds, other sources | 86 | 21 |
| (Ma et al., 2009) | A=1993-1994,  B=2005-2006 | Pediatric patients: period A included pharyngitis, scarlet fever, impetigo, otitis media, nephritis, erysipelas, cellulitis; period B included pharyngitis, scarlet fever, impetigo, erysipelas, bronchitis, colpitis, nephritis, urticaria, pleural effusion, sepsis, psoriasis. | China | In period A, throat swabs, pus, ear swabs. In period B, throat swabs, puss, vulvar secretions, pleural effusion, and blood. | 359 | A=24,  B=9 |
| (Feng et al., 2010) | 2005-2008 | Pediatric patients who had GAS infection | Beijing, Chongqing, Shenzhen | Throat swabs, pus, vulvar secretions, pleural effusion, blood | 319 | 11 |
| (Chang et al., 2011) | 2007-2008 | Outpatients diagnosed with pharyngitis in 4 children's hospitals in Beijing, Shanghai, Shenzhen, and Chongqing | Beijing, Shanghai, Chongqing, Shenzhen | Throat swab | 185 | 13 |
| (Liang et al., 2012) | 2005-2008 | Children with pharyngitis, scarlet fever, or impetigo; carriers aged 7–14 years who were healthy but were found to carry S. *pyogenes* in pharyngeal samples. | Beijing, Shanghai, Chongqing, Shenzhen | Throat swab, skin swab | 466 | 19 |
| (You et al., 2013) | 2011 | Pediatric patients with scarlet fever or tonsillopharyngitis | Beijing, Heilongjiang, Tianjin | Throat swab | 74 | 4 |
| (Ho et al., 2003) | 1995-1998 | Patients with severe systemic GAS infections (invasive group); patients with minor skin and throat infections (noninvasive group). | Hong Kong | Sterile sites or non-sterile sites | 107 | 32 |
| (Chan et al., 2009) | 2005.10-2008.04 | Most patients suffering from invasive S. *pyogenes* infections | Hong Kong | Sterile sites or non-sterile sites | 285 | 46 |
| (Luk et al., 2012) | 2011.01-06 | Scarlet fever patients | Hong Kong | Mostly throat swab specimens | 90 | 6 |
| (Yan et al., 2003) | 1993-2002 | Scarlet fever patients | Taiwan | Throat swab | 77 | 3 |
| (Huang et al., 2014) | 1998-2010 | GAS isolates were collected from hospitals participating the Taiwan Surveillance of Antimicrobial Resistance (TSAR) biennially. | Taiwan | Mostly from the respiratory tract, blood, abscess/pus, or other specimen sources. | 127 | 20 |
| (Chen et al., 2007) | 2001-2002 | Scarlet fever patients | Taiwan | Throat swab | 830 | 21 |
| (Lin et al., 2011) | 2005-2007 | Patients with skin and soft tissue infections (SSTIs) caused by GAS | Taiwan | Sterile sites or non-sterile sites | 73 | 18 |
| (Chiang-Ni et al., 2021) | 2008-2019 | GAS isolates collected at the LinKou Chang Gung Memorial Hospital (Taiwan). | Taiwan | Sterile tissues (ascites, blood, deep tissue, pleural fluid, and synovial fluid) | 220 | 34 |
| (Chiou et al., 2004) | 1996-1999 | Scarlet fever patients | Taiwan | NA | 179 | 9 |
| (Chiou et al., 2009) | 2000-2006 | Scarlet fever patients | Taiwan | Throat swab | 1218 | 23 |
| (Tsai et al., 2021) | 2000-2019 | Children <18 years who presented with signs and symptoms of an acute upper respiratory tract infection (URTI) from outpatient clinics, emergency departments and inpatients. | Taiwan | Throat swab | 320 | 16 |
| (Lau et al., 2003) | 2003 | The first case of a primary GAS psoas abscess in China | Hong Kong | Pus | 1 | 1 |
| (Zheng et al., 2009) | 2005 | Acute glomerulonephritis (AGN) patients, pharyngitis patients, carriers with GAS | Guizhou | Throat swab | 27 | 11 |
| (Huang et al., 2006) | 2006 | Acute rheumatic fever patient | Guangdong | Throat swab | 1 | 1 |
| (Dong et al., 2007) | 2006 | Scarlet fever patient | Zhejiang | Throat swab | 1 | 1 |
| (Liu et al., 2014) | 2012 | The first outbreak of a single clone GAS (*emm-*type 89) tonsillopharyngitis in China | Beijing | Pharyngeal swab | 1 | 1 |
| (Xu et al., 2016) | 2012 | Scarlet fever patient | Zhejiang | Throat swab | 2 | 2 |
| (Chen et al., 2017) | 2013 | Patients with GAS infection and healthy carriers with GAS | Shanghai | Throat swab | 10 | 3 |
| (Wang et al., 2016) | 2015 | The first case of severe streptococcal toxic shock syndrome (STSS) caused by *emm*89 *Streptococcus pyogenes* | Beijing | Skin swab | 1 | 1 |
| (Ning et al., 2017) | 2017 | A case with STSS caused by *emm*1.1 *Streptococcus pyogenes* | Hunan | Skin swab | 1 | 1 |

(Note: NA=not available)

6. Citations for all sources used for data analysis:

1. Bai, A., Liu, L., Chai, Z., Liu, Z., and Shan, X. (2019). Molecular typing of β-haemolytic group A *Streptococcus* isolates in Jinan of Shandong, 2012–2018. *DISEASE SURVEILLANCE*. doi: 10.3784/j.issn.1003-9961.2019.05.006.
2. Chan, J.C., Chu, Y.W., Chu, M.Y., Cheung, T.K., and Lo, J.Y. (2009). Epidemiological analysis of *Streptococcus pyogenes* infections in Hong Kong. *Pathology* 41(7)**,** 681-686. doi: 10.3109/00313020903257723.
3. Chang, H., Shen, X., Huang, G., Fu, Z., Zheng, Y., Wang, L., et al. (2011). Molecular analysis of *Streptococcus pyogenes* strains isolated from Chinese children with pharyngitis. *Diagn Microbiol Infect Dis* 69(2)**,** 117-122. doi: 10.1016/j.diagmicrobio.2010.09.011.
4. Chen, M., Wang, W., Tu, L., Zheng, Y., Pan, H., Wang, G., et al. (2017). An *emm*5 group A streptococcal outbreak among workers in a factory manufacturing telephone accessories. *Front Microbiol* 8**,** 1156. doi: 10.3389/fmicb.2017.01156.
5. Chen, Y.Y., Huang, C.T., Yao, S.M., Chang, Y.C., Shen, P.W., Chou, C.Y., et al. (2007). Molecular epidemiology of group A *Streptococcus* causing scarlet fever in northern Taiwan, 2001-2002. *Diagn Microbiol Infect Dis* 58(3)**,** 289-295. doi: 10.1016/j.diagmicrobio.2007.01.013.
6. Chen, Z., Xie, Y., and Deng, Q. (2008). *Emm* genotyping of 87 strains of group A streptococci infections in children in Guangzhou. *Guangdong Medical Journal*. doi: 10.13820/j.cnki.gdyx.2008.04.052.
7. Chiang-Ni, C., Liu, Y.S., Lin, C.Y., Hsu, C.Y., Shi, Y.A., Chen, Y.M., et al. (2021). Incidence and effects of acquisition of the phage-encoded *ssa* superantigen gene in invasive group A *Streptococcus*. *Front Microbiol* 12**,** 685343. doi: 10.3389/fmicb.2021.685343.
8. Chiou, C.S., Liao, T.L., Wang, T.H., Chang, H.L., Liao, J.C., and Li, C.C. (2004). Epidemiology and molecular characterization of *Streptococcus pyogenes* recovered from scarlet fever patients in central Taiwan from 1996 to 1999. *J Clin Microbiol* 42(9)**,** 3998-4006. doi: 10.1128/jcm.42.9.3998-4006.2004.
9. Chiou, C.S., Wang, Y.W., Chen, P.L., Wang, W.L., Wu, P.F., and Wei, H.L. (2009). Association of the shuffling of *Streptococcus pyogenes* clones and the fluctuation of scarlet fever cases between 2000 and 2006 in central Taiwan. *BMC Microbiol* 9**,** 115. doi: 10.1186/1471-2180-9-115.
10. Dong, H., Song, Q., Lin, H., Jin, C., and Xu, G. (2007). "Study of genotypic characteristics and virulent genes of group A *Streptococcus* isolates recovered during a scarlet fever outbreak", in: *Zhejiang Medical Virology, medical microbiology and immunology academic annual meeting in 2007* *Zhejiang, China*, 110-114.
11. Feng, L., Lin, H., Ma, Y., Yang, Y., Zheng, Y., Fu, Z., et al. (2010). Macrolide-resistant *Streptococcus pyogenes* from Chinese pediatric patients in association with Tn916 transposons family over a 16-year period. *Diagn Microbiol Infect Dis* 67(4)**,** 369-375. doi: 10.1016/j.diagmicrobio.2010.03.014.
12. Ho, P.L., Johnson, D.R., Yue, A.W., Tsang, D.N., Que, T.L., Beall, B., et al. (2003). Epidemiologic analysis of invasive and noninvasive group A streptococcal isolates in Hong Kong. *J Clin Microbiol* 41(3)**,** 937-942. doi: 10.1128/jcm.41.3.937-942.2003.
13. Hong, J., Tan, Z., Xu, K., Huang, H., and Li, C. (2021). Antimicrobial resistance analysis and molecular features of group A streptococci from Jiangsu Province, China. *Chinese Journal of Health Laboratory Technology*.
14. Huang, C.Y., Lai, J.F., Huang, I.W., Chen, P.C., Wang, H.Y., Shiau, Y.R., et al. (2014). Epidemiology and molecular characterization of macrolide-resistant *Streptococcus pyogenes* in Taiwan. *J Clin Microbiol* 52(2)**,** 508-516. doi: 10.1128/JCM.02383-13.
15. Huang, J., Yu, B., and Gu, J. (2006). A strain of group A β-hemolytic *Streptococcus* isolated from the throat of a patient with rheumatic fever. *Journal of Practical Medicine* (09)**,** 1058. doi: 10.3969/j.issn.1006-5725.2006.09.060
16. Ji, L., Yu, S., Liu, Y., and Zhang, X. (2012). The characteristics of *Streptococus pyogens* from Chinese children with impetigo between 2003 and 2008. *Chinese Journal of Practical Pediatrics.* doi: CNKI:SUN:ZSEK.0.2012-06-027
17. Jing, H.B., Ning, B.A., Hao, H.J., Zheng, Y.L., Chang, D., Jiang, W., et al. (2006). Epidemiological analysis of group A streptococci recovered from patients in China. *J Med Microbiol* 55(Pt 8)**,** 1101-1107. doi: 10.1099/jmm.0.46243-0.
18. Lau, S.K., Woo, P.C., Yim, T.C., To, A.P., and Yuen, K.Y. (2003). Molecular characterization of a strain of group A *Streptococcus* isolated from a patient with a psoas abscess. *J Clin Microbiol* 41(10)**,** 4888-4891. doi: 10.1128/jcm.41.10.4888-4891.2003.
19. Li, G., Tan, Z., Li, Y., Dong, T., and Ou, G. (2009). Pathogenic group A streptococcal *emm* gene sequence typing study in Xinxing Guangdong Province. *International Medicine & Health Guidance News*. doi: 10.3760/cma.j.issn.1007-1245.2009.24.001
20. Li, H., Zhou, L., Zhao, Y., Ma, L., Liu, X., and Hu, J. (2020a). Molecular epidemiology and antimicrobial resistance of group A *Streptococcus* recovered from patients in Beijing, China. *BMC Infect Dis* 20(1)**,** 507. doi: 10.1186/s12879-020-05241-x.
21. Li, H., Zhou, L., Zhao, Y., Ma, L., Xu, J., Liu, Y., et al. (2020b). Epidemiological analysis of group A *Streptococcus* infections in a hospital in Beijing, China. *Eur J Clin Microbiol Infect Dis* 39(12)**,** 2361-2371. doi: 10.1007/s10096-020-03987-5.
22. Li, M., Luo, Z., He, G., and Fu, Z. (2010). *Emm* type, macrolide-resistance genes and superantigen gene profile among GAS isolate from children with pharyngitis in Chongqing. *Journal of Chongqing Medical University*. doi: 10.13406/j.cnki.cyxb.2010.03.037.
23. Li, Y., Wang, W., Lou, C., and Gao, J. (2018). A pathogen study of group A *Streptococcus* causing acute post-streptococcal glomerulonephritis. *Journal of Pathogen Biology*. doi: 10.13350/j.cjpb.180722.
24. Liang, Y., Chang, H., Shen, X., Yu, S., Yuan, L., and Yang, Y. (2010). Relationship between *emm* typing and superantigen genes *speA* and *speC* of *Streptococcus Pyogenes* isolated from children in Beijing. *Journal of Applied Clinical Pediatrics*. doi: CNKI:SUN:SYQK.0.2010-22-005
25. Liang, Y., Liu, X., Chang, H., Ji, L., Huang, G., Fu, Z., et al. (2012). Epidemiological and molecular characteristics of clinical isolates of *Streptococcus pyogenes* collected between 2005 and 2008 from Chinese children. *J Med Microbiol* 61(Pt 7)**,** 975-983. doi: 10.1099/jmm.0.042309-0.
26. Lin, J.N., Chang, L.L., Lai, C.H., Lin, H.H., and Chen, Y.H. (2011). Clinical and molecular characteristics of invasive and noninvasive skin and soft tissue infections caused by group A *Streptococcus*. *J Clin Microbiol* 49(10)**,** 3632-3637. doi: 10.1128/jcm.00531-11.
27. Liu, Y.M., Zhao, J.Z., Li, B.B., Yang, J.Y., Dong, X.G., Zhang, J.J., et al. (2014). A report on the first outbreak of a single clone group A *Streptococcus* (*emm*-type 89) tonsillopharyngitis in China. *J Microbiol Immunol Infect* 47(6)**,** 542-545. doi: 10.1016/j.jmii.2013.08.011.
28. Liu, Z. (2015). *Molecular typing and resistance of group A Streptococcus in Shandong Province, 2013-2014.* Masters, Shandong University.
29. Luk, E.Y., Lo, J.Y., Li, A.Z., Lau, M.C., Cheung, T.K., Wong, A.Y., et al. (2012). Scarlet fever epidemic, Hong Kong, 2011. *Emerg Infect Dis* 18(10)**,** 1658-1661. doi: 10.3201/eid1810.111900.
30. Ma, Y., Yang, Y., Huang, M., Wang, Y., Chen, Y., Deng, L., et al. (2009). Characterization of *emm* types and superantigens of *Streptococcus pyogenes* isolates from children during two sampling periods. *Epidemiol Infect* 137(10)**,** 1414-1419. doi: 10.1017/s0950268809002118.
31. Ning, X., Zhu, H., Qian, C., Deng, L., and Xie, X. (2017). Analysis of toxic shock syndrome caused by *Streptococcus pyogenes*. *Lab Med Clin* 14(23)**,** 3444-3446. doi: 10.3969/j.issn.1672-9455.2017.23.008.
32. Ouyang, L., Wang, X., Song, C., Chen, M., and Zhang, Y. (2021). Distinctive colony morphology categorized by *emm* genotypes of *Streptococcus pyogenes*. *Fudan University Journal of Medical Sciences* doi: 10.3969/j.issn.1672-8467.2021.04.022.
33. Shen, Y., Cai, J., Davies, M.R., Zhang, C., Gao, K., Qiao, D., et al. (2018). Identification and characterization of fluoroquinolone non-susceptible *Streptococcus pyogenes* clones harboring tetracycline and macrolide resistance in Shanghai, China. *Front Microbiol* 9**,** 542. doi: 10.3389/fmicb.2018.00542.
34. Tan, X., Liu, M., Yang, Y., Li, B., and Kang, M. (2019). Epidemiological characteristics of scarlet fever in Guangdong province, 2005-2017. *DISEASE SURVEILLANCE*. doi: 10.3784/j.issn.1003-9961.2019.05.010.
35. Tsai, W.C., Shen, C.F., Lin, Y.L., Shen, F.C., Tsai, P.J., Wang, S.Y., et al. (2021). Emergence of macrolide-resistant *Streptococcus pyogenes* *emm*12 in southern Taiwan from 2000 to 2019. *J Microbiol Immunol Infect* 54(6)**,** 1086-1093. doi: 10.1016/j.jmii.2020.08.019.
36. Wang, L., Cao, H., Sun, T., Ma, M., Cui, F., Fang, M., et al. (2020). Epidemiological characteristics and pathogenic surveillance of scarlet fever in Zibo, 2015-2019. *Modem Preventive Medicine* 47(21)**,** 3998-4001.
37. Wang, P., Yang, Q., Zhou, X., Zhao, X., Wang, Y., Wang, H., et al. (2016). Clinical analysis of adults with toxic shock syndrome induced by *Streptococcus pyogenes*. *Chin J Nosocomiol* 26(10)**,** 2251-2253+2259. doi: 10.11816/cn.ni.2016-160887.
38. Xu, Y., Luo, Y., Sun, Y., and Chen, Y. (2016). Etiological detection and molecular characteristics analysis of the outbreaks of scarlet fever. *Chin J Health Lab Tec* 26(04)**,** 553-555. doi: CNKI:SUN:ZWJZ.0.2016-04-035
39. Yan, J.J., Liu, C.C., Ko, W.C., Hsu, S.Y., Wu, H.M., Lin, Y.S., et al. (2003). Molecular analysis of group A streptococcal isolates associated with scarlet fever in southern Taiwan between 1993 and 2002. *J Clin Microbiol* 41(10)**,** 4858-4861. doi: 10.1128/jcm.41.10.4858-4861.2003.
40. Yang, Z., and Wang, J. (2020). Epidemiology and drug resistance of *Streptococcus pyogenes* in scarlet fever. *Chinese Journal of Woman and Child Health Research* 31(01)**,** 35-38. doi: 10.3969/j.issn.1673-5293.2020.01.008.
41. Yin, J., Zhang, W., Yang, D., Dong, X., and Li, L. (2019). Characteristics of antibiotic resistance in group A *Streptococcus* strains isolated from children with scarlet fever. *Chinese Journal of Microbiology and Immunology*. doi: 10.3760/cma.j.issn.0254-5101.2019.01.006.
42. You, Y., Peng, X., Yang, P., Wang, Q., and Zhang, J. (2020). 8-year M type surveillance of *Streptococcus pyogenes* in China. *Lancet Infect Dis* 20(1)**,** 24-25. doi: 10.1016/s1473-3099(19)30694-2.
43. You, Y.H., Song, Y.Y., Yan, X.M., Wang, H.B., Zhang, M.H., Tao, X.X., et al. (2013). Molecular epidemiological characteristics of *Streptococcus pyogenes* strains involved in an outbreak of scarlet fever in China, 2011. *Biomed Environ Sci* 26(11)**,** 877-885. doi: 10.3967/bes2013.016.
44. Yu, D., Liang, Y., Lu, Q., Meng, Q., Wang, W., Huang, L., et al. (2021). Molecular characteristics of *Streptococcus pyogenes* isolated from Chinese children with different diseases. *Front Microbiol* 12**,** 722225. doi: 10.3389/fmicb.2021.722225.
45. Zeng, J., Liu, A., Fang, D., Xiang, H., and Tian, G. (2016). Research the epidemic situation and *emm* genotyping of children infected with pathogenic group A *Streptococcus* in Shenzhen Baoan District. *Chinese Journal of Birth Health & Heredity* 24. doi: 10.13404/j.cnki.cjbhh.2016.07.055.
46. Zhang, Y., Yu, H., Wang, X., He, Y., and Yan, H. (2019). Molecular epidemiological characteristics of *Streptococcus pyogenes* causing scarlet fever and angina in children. *Chinese Journal of Microbiology and Immunology*. doi: 10.3760/cma.j.issn.0254-5101.2019.11.003.
47. Zheng, M.H., Jiao, Z.Q., Zhang, L.J., Yu, S.J., Tang, G.P., Yan, X.M., et al. (2009). Genetic analysis of group A *Streptococcus* isolates recovered during acute glomerulonephritis outbreaks in Guizhou Province of China. *J Clin Microbiol* 47(3)**,** 715-720. doi: 10.1128/jcm.00747-08.

7.Studies excluded due to duplication of data set.

1. Chang, H., Shen, X., Fu, Z., Liu, L., Shen, Y., Liu, X., et al. (2010). Antibiotic resistance and molecular analysis of *Streptococcus pyogenes* isolated from healthy schoolchildren in China. *Scand J Infect Dis* 42(2)**,** 84-89. doi: 10.3109/00365540903321598.
2. Chen, M., Cai, J., Davies, M.R., Li, Y., Zhang, C., Yao, W., et al. (2020). Increase of *emm*1 isolates among group A *Streptococcus* strains causing scarlet fever in Shanghai, China. *Int J Infect Dis* 98**,** 305-314. doi: 10.1016/j.ijid.2020.06.053.
3. Chen, M., Yao, W., Wang, X., Li, Y., Chen, M., Wang, G., et al. (2012). Outbreak of scarlet fever associated with *emm*12 type group A *Streptococcus* in 2011 in Shanghai, China. *Pediatr Infect Dis J* 31(9)**,** e158-162. doi: 10.1097/INF.0b013e31825874f3.
4. Cui, J., Yuan, M., Hu, X., Jin, B., Ji, Y., and Li, D. (2014). Genotyping of *emm* gene in group A hemolytic *Streptococcus* from children in Xicheng district of Beijing from 2011 to 2013. *Chin J Health Lab Tec* 24(14)**,** 2048-2050+2056. doi: CNKI:SUN:ZWJZ.0.2014-14-026
5. Gao, K., Chen, M., Han, Q., and Wu, W. (2017). Analysis the epidemiology and drug sensitivity of group A *Streptococcus* strains isolated from children in partial areas of Shanghai. *Chin J Lab Med* 40(5)**,** 362-366. doi: 10.3760/cma.j.issn.1009-9158.2017.05.007.
6. Li, J., Liu, S., Peng, X., Yang, P., Zhang, D., Wu, S., et al. (2012). Study on types of M protein gene in group A *Streptococcus* isolated from children in Beijing, 2011. *Chin J Prev Med* (12)**,** 1107-1111. doi: 10.3760/cma.j.issn.0253-9624.2012.12.013.
7. Li, Y., Peng, X., Gao, P., and Yang, P. (2019). Genotying research of M protein gene in group A *Streptococcus* from children in Dongcheng District, Beijing from 2011-2017. *J Med Pest Control* 35(10)**,** 958-960. doi: 10.7629/yxdwfz201910011.
8. Liang, Y., Shen, X., Huang, G., Wang, C., Shen, Y., and Yang, Y. (2008). Characteristics of *Streptococcus pyogenes* strains isolated from Chinese children with scarlet fever. *Acta Paediatr* 97(12)**,** 1681-1685. doi: 10.1111/j.1651-2227.2008.00983.x.
9. Liang, Y., Yang, Y., Yu, S., Yuan, L., Yao, K., Ren, S., et al. (2014). Molecular biological characteristic of *Streptococcus pyogenes* causing children tonsillitis in Yangfangdian community Haidian District of Beijing from 2011-2013. *Chin J Appl Clin Pediatr* 29(16)**,** 1220-1223. doi: 10.3760/cma.j.issn.2095-428X.2014.16.007.
10. Liu, Y., Yang, P., Wu, S., Pan, Y., Lu, G., Zhang, D., et al. (2017). The *emm* genotype of group A *Streptococcus* isolated from clinical infected children, Beijing. *Int J Lab Med* 38(24)**,** 3368-3370. doi: 10.3969/j.issn.1673-4130.2017.24.004.
11. Lu, G., Zhang, D., Zhao, J., Liu, Y., Guo, J., Wu, S., et al. (2015). Study on the superantigen gene profiles of group A *Streptococcus* isolated from children in Beijing, 2014. *Chin J Prev Med* 49(11)**,** 988-992. doi: 10.3760/cma.j.issn.0253-9624.2015.11.012.
12. Ma, C., Peng, X., Wu, S., Zhang, D., Lu, G., Pan, Y., et al. (2018). Study on the super-antigen genes of group A *Streptococcus* strains isolated from patients with scarlet fever and pharyngeal infection, in Beijing, 2015-2017. *Chin J Epidemiol* 39(10)**,** 1375-1380. doi: 10.3760/cma.j.issn.0254-6450.2018.10.016.
13. Ma, Y., Yang, Y., Yu, S., Yao, K., Yuan, L., and Shen, X. (2009). *Emm* types and superantigen analysis of *Streptococcus pyogenes* isolated from Chinese children. *Basic & Clinical Medicine* 29(11)**,** 1166-1169. doi: 10.16352/j.issn.1001-6325.2009.11.009.
14. Peng, X., Liu, S., Yang, P., Li, J., Zhang, D., Cui, S., et al. (2014). Study on the distribution of superantigen of group A *Streptococcus* isolated from children in Beijing, 2011. *Chin J Epidemiol* 35(3)**,** 299-302. doi: 10.3760/cma.j.issn.0254-6450.2014.03.018.
15. Peng, X., Yang, P., Wu, S., Lu, G., Shi, W., Zhao, J., et al. (2015). *Emm* types of mutation in scarlet-fever-related group A streptococcal, among children in Beijing, 2011-2014. *Chin J Epidemiol* 36(12)**,** 1397-1400. doi: 10.3760/cma.j.issn.0254-6450.2015.12.018.
16. Qiao, D., Chen, M., Zhu, Y., Fan, Q., and Zhou, M. (2016). Molecular epidemiological analysis of *Streptococcus pyogenes* isolated from children in Jiading, Shanghai. *Laboratory Medicine* 31(12)**,** 1061-1065. doi: 10.3969/j.issn.1673-8640.2016.12.011
17. Wang, B., Yang, P., Peng, X., Zhang, D., Cui, S., Zhao, J., et al. (2015a). The distribution of the *emm* types and antimicrobial susceptibility to group A *Streptococcus* in children of Beijing, 2014. *Chin J Appl Clin Pediatr* 30(22)**,** 1697-1700. doi: 10.3760/cma.j.issn.2095-428X.2015.22.005.
18. Wang, H.B., Song, Y.Y., You, Y.H., Wang, H.W., Han, Q.H., Zhao, J.H., et al. (2013). Molecular epidemiological analysis of group A streptococci isolated from children in Chaoyang District of Beijing, 2011: *emm* types, virulence factor genes and erythromycin resistant genes. *Biomed Environ Sci* 26(9)**,** 782-784. doi: 10.3967/0895-3988.2013.09.012.
19. Wang, Z.-e., Feng, H., Yu, H., Tang, X., and Dong, X. (2015b). Study on *emm* genotyping and drug-resistance gene of group A *Streptococcus* in Fengtai, Beijing (2011-2014). *J of Pub Health and Prev Med* 26(05)**,** 22-24. doi: CNKI:SUN:FBYF.0.2015-05-006
20. Wu, S., Peng, X., Ma, C., Zhang, D., Lu, G., Pan, Y., et al. (2015). Study on the relationship between M protein gene-types and superantigen genes of group A *Streptococcus* strains isolated from scarlet fever in Beijing. *Chin J Infect Dis* 33(10)**,** 611-614. doi: 10.3760/cma.j.issn.1000-6680.2015.10.006.
21. Xiao, H., Yin, X., Zhang, H., and Liu, Q. (2016). Analysis of the prevalence status and M protein gene classification of A group pathogenic *Streptococcus* infection of children in Shenzhen Area. *J Mod Lab Med* 31(06)**,** 51-54. doi: 10.3969/j.issn.1671-7414.2016.06.014.
22. Yang, P., Peng, X., Zhang, D., Wu, S., Liu, Y., Cui, S., et al. (2013). Characteristics of group A *Streptococcus* strains circulating during scarlet fever epidemic, Beijing, China, 2011. *Emerg Infect Dis* 19(6)**,** 909-915. doi: 10.3201/eid1906.121020.
23. Yin, J., Zhang, W., Yang, D., Li, L., and Dong, X. (2018). Etiological characteristics of *Streptococcus pyogenes* isolated from children with scarlet fever in Tianjin from 2012 to 2016. *Chin J Prev Med* 52(10)**,** 1045-1049. doi: 10.3760/cma.j.issn.0253-9624.2018.10.015.
24. Zhang, D., Yang, P., Wu, S., Zhao, J., Lu, G., Guo, J., et al. (2015). M protein gene of group A *Streptococcus* isolated from children with pharyngeal infection in Beijing, 2011-2014. *DISEASE SURVEILLANCE* 30(11)**,** 917-921. doi: 10.3784/j.issn.1003-9961.2015.11.007.
25. Zheng, P.X., Chan, Y.C., Chiou, C.S., Chiang-Ni, C., Wang, S.Y., Tsai, P.J., et al. (2015). Clustered regularly interspaced short palindromic repeats rre *emm* type-specific in highly prevalent group A streptococci. *PLoS One* 10(12)**,** e0145223. doi: 10.1371/journal.pone.0145223.

8. The number and proportion of the 10 most common *emm* genotypes strains of GAS in mainland China in 1990s, 2000s and 2010s.

| ***Emm* types** | **1990s** | **2000s** | **2010s** |
| --- | --- | --- | --- |
| 12 | 14(10.22%) | 799(45.89%) | 4115(59.28%) |
| 1 | 24(17.52%) | 619(35.55%) | 2420(34.86%) |
| 22 | 1(0.73%) | 66(3.79%) | 43(0.62%) |
| 4 | 19(13.87%) | 28(1.61%) | 29(0.42%) |
| 3 | 36(26.28%) | 8(0.46%) | 26(0.37%) |
| 75 | - | 12(0.69%) | 45(0.65%) |
| 6 | 9(6.57%) | 11(0.63%) | 29(0.42%) |
| 18 | 5(3.65%) | 37(2.13%) | 5(0.07%) |
| 89 | 2(1.46%) | - | 39(0.56%) |
| 110 | - | 14(0.8%) | 22(0.32%**)** |

9. The number and proportion of the 10 most common *emm* genotypes strains of GAS in Hong Kong in 1990s, 2000s and 2010s.

| ***Emm* types** | **1990s (%)** | **2000s (%)** | **2010s (%)** |
| --- | --- | --- | --- |
| 12 | 22(20.56%) | 61(21.33%) | 70(77.78%) |
| 1 | 16(14.95%) | 61(21.33%) | 14(15.56%) |
| 4 | 10(9.35%) | 40(13.99%) | 2(2.22%) |
| 2 | - | 21(7.34%) | 1(1.11%) |
| 22 | 3(2.8%) | 16(5.59%) | 2(2.22%) |
| 58 | 11(10.28%) | 2(0.7%) | - |
| 77 | 3(2.8%) | 7(2.45%) | - |
| 104 | - | 9(3.15%) | - |
| 49 | 5(4.67%) | 3(1.05%) | - |
| 87 | 2(1.87%) | 6(2.1%) | - |

10. The number and proportion of the 10 most common *emm* genotypes strains of GAS in Taiwan in 1990s, 2000s and 2010s.

| ***Emm* types** | **1990s (%)** | **2000s (%)** | **2010s (%)** |
| --- | --- | --- | --- |
| 12 | 79(28.21%) | 872(35.69%) | 151(47.04%) |
| 4 | 109(38.93%) | 537(21.98%) | 5(1.56%) |
| 1 | 52(18.57%) | 514(21.04%) | 42(13.08%) |
| 6 | 2(0.71%) | 185(7.57%) | - |
| 22 | 14(5.00%) | 88(3.6%) | 12(3.74%) |
| 11 | - | 32(1.31%) | 4(1.25%) |
| 106 | - | 33(1.35%) | - |
| 102 | - | 13(0.53%) | 12(3.74%) |
| 113 | 1(0.36%) | - | 20(6.23%) |
| 89 | 1(0.36%) | 8(0.33%) | 8(2.49%) |
